# Telehealth and Collaboratively Delivered Dialectical Behaviour Therapy: An Opportunity for Increasing Access to Effective Treatment for People With Borderline Personality Disorder Living in Rural Areas

**DOI:** 10.1111/ajr.70036

**Published:** 2025-04-03

**Authors:** Carla J. Walton, Sharleen Gonzalez, Anna Dunbar, Katie McGill

**Affiliations:** ^1^ Hunter New England Mental Health Service Newcastle New South Wales Australia; ^2^ School of Medicine and Public Health, College of Health, Medicine and Wellbeing University of Newcastle Callaghan New South Wales Australia; ^3^ Hunter Medical Research Institute (HMRI) New Lambton New South Wales Australia; ^4^ Community Mental Health Drug and Alcohol Murrumbidgee Local Health District Wagga Wagga New South Wales Australia

**Keywords:** borderline personality disorder, dialectical behaviour therapy, rural, telehealth

## Abstract

**Aims:**

The aim of this commentary is to consider how telehealth and a collaborative model of service delivery may offer a way of making Dialectical Behaviour Therapy available in rural areas.

**Context:**

Dialectical Behaviour Therapy (DBT) is an effective treatment for Borderline Personality Disorder (BPD). However, there are many barriers to making this sort of therapy available within routine care, particularly in rural areas.

**Approach:**

This commentary provides a summary of the literature relevant to the role that telehealth could play in increasing access to DBT. A new model of care could utilise telehealth services to deliver comprehensive DBT treatment to people with BPD living in rural areas in partnership with community mental health services.

**Conclusion:**

Telehealth and collaborative models of Dialectical Behaviour Therapy delivery should be further investigated, especially to meet the needs of rural mental health care.

**Summary**

- Borderline Personality Disorder (BPD) is a mental illness with prevalence estimates of 1%–2% of the general population, 10% of psychiatric outpatients and 25% of inpatients meet diagnostic criteria for BPD.
- Service availability of comprehensive BPD treatment is lacking in rural areas in Australia, despite great need.
- Training needs to be improved in rural areas to equip clinicians to treat BPD.
- Dialectical Behaviour Therapy (DBT) is one of the most researched effective treatments for BPD.
- Clinician training in DBT has been associated with improved therapist attitudes towards consumers with BPD and improved confidence in the treatment of this population.
- There is emerging evidence that DBT can be delivered via telehealth. Using telehealth may be a way to address some of the barriers to DBT being available in rural settings.
- A model of service delivery for rural settings involving a mix of telehealth and face‐to‐face delivery methods across service centres would (1) reduce inequity of mental health care for people with BPD in rural areas and (2) enhance multi‐disciplinary collaboration and assist rural clinicians to feel a greater sense of connectedness through regular meetings and consultation.

## 1 Background: Borderline Personality Disorder—An Underserved Clinical Population in Rural Areas

Borderline Personality Disorder (BPD) is a mental illness, characterised by chronic emotion dysregulation, self‐injury and suicidal behaviour. Prevalence estimates suggest 1%–2% of the general population meet diagnostic criteria for BPD, 10% of psychiatric outpatients and 25% of inpatients meet diagnostic criteria for BPD [1, 2].

BPD is associated with high emotional, social and occupational burden and suffering for patients and their families [3]. There is high stigma associated with a BPD diagnosis [4]. Consumers living in rural areas have described feeling that they needed to hide their diagnosis and were reluctant to seek help due to community‐based stigma [5]. Given the presence of self‐injury and suicidal behaviour in this population, this understandable reluctance to seek support could be life threatening.

BPD is also one of the most expensive psychiatric disorders for health systems [6]. There is extensive use of treatment services with this population, requiring multiple repeated treatments, including urgent interventions in emergency departments for suicide attempts or self‐injurious behaviour.

Despite the acuity and frequent contact, there are many studies showing that clinicians are reluctant to work with people with BPD due to its complex and challenging nature [7] and because they believe they lack the skill and confidence to be effective [8]. This lines up with the consumer perspective. In a study exploring the management of BPD in Australia, consumers with a diagnosis of BPD emphasised that training needed to be improved in rural areas to equip clinicians to understand what care is needed [9].

## 2 Dialectical Behaviour Therapy: An Effective Treatment

Dialectical Behaviour Therapy (DBT) is one of the most researched effective treatments available for BPD. DBT posits that BPD is a disorder of emotion dysregulation and it was developed as a treatment for individuals with a diagnosis of BPD with co‐morbid mental health disorders at high risk for suicide [10]. There is extensive research showing that when delivered face to face, DBT leads to a reduction of non‐suicidal and suicidal self‐injurious behaviour, hospital admissions and a variety of other outcomes [11]. Standard DBT includes four modes of treatment: consumers attend (1) weekly individual therapy sessions, (2) a weekly skills training group, (3) have access to phone coaching and (4) therapists attend a weekly consultation meeting. DBT is typically delivered by clinicians in a single team that is physically co‐located. Whilst time intensive, DBT has been demonstrated to be cost‐effective by virtue of hospital cost savings alone for people with BPD [12]. In addition, clinician training and involvement in DBT have been associated with improved therapist attitudes towards consumers with BPD and improved confidence in the effectiveness of DBT for this population group [13].

## 3 Dialectical Behaviour Therapy: Limited Availability in Rural Areas

Service availability of any effective BPD treatment, let alone DBT, is a major issue for individuals living in rural areas in Australia [14]. Mental health outcomes for rural people are persistently poorer when compared to metropolitan areas despite similar prevalence [15]. People with BPD living in rural Australia continue reporting multiple barriers including lack of local services, long wait lists for individual and group programmes, unaffordable therapies and long commutes to city‐based services [9]. The physical distance between individuals with BPD and effective healthcare is a significant barrier, with cited issues including ‘lack of transportation, poor weather or road conditions or financial restrictions related to paying for transportation’ [5]. The benefits of receiving a diagnosis of BPD such as informed treatment planning and provision are reduced in rural areas by the challenges of stigma and lack of treatment options [16].

Staffing issues and high turnover of mental health staff that are consistently reported in rural settings are thought to be a consequence of additional stressors such as higher and more complex workloads, professional isolation and reduced resources [17]. This results in less continuity of care and less experienced clinicians. Community mental health services have historically been designed for delivering treatment relevant to schizophrenia and other psychotic disorders [18], leaving the complex needs of people with BPD in an unaddressed gap, particularly in rural areas.

A study of rural residents' experience living with BPD [5] reported that some individuals had to relocate to urban areas to receive appropriate treatment. That has also been our own experience. These sorts of experiences highlight that we need to be more creative in solving the issue of treatment accessibility, as the current approaches to BPD care in rural areas do not appear to be effective. It is reasonable that people in rural areas can remain in their communities to receive treatment, yet that is often not the situation due to the significant gap in care that exists for people with BPD in rural communities.

In summary, whilst there is extensive research showing the effectiveness of DBT, it is a challenging treatment to deliver in rural settings given the resource intensiveness of the intervention alongside the day‐to‐day demands on clinicians in rural settings. A small study completed within community mental health services in regional South Australia found a group DBT skills program was effective in improving BPD‐related symptoms and reducing presentations and hospitalisations of BPD participants [19]. This is promising as another option for increasing access to DBT‐informed treatments. However, in the rural areas of our own health services, DBT group programs have been established and run for short periods of time, but have been hard to sustain and hence have ended up ceasing.

## 4 Telehealth as Part of the Solution?

Two solutions outlined in the Orange Declaration to address poor rural mental health outcomes [15] were to use telehealth in an integrated way rather than in isolation and to develop new rural workforce models. Telehealth solutions have been proposed for multiple reasons including as a way to use resources efficiently, provide supportive networks to clinicians and bridge the enormous distances between patients and clinicians to improve service access. These issues are particularly relevant in a country the size of Australia with approximately 1/3 of the population living in rural or remote locations [20].

There is a large amount of evidence that there are no differences between treatment outcomes delivered via telehealth compared to face‐to‐face delivery for a range of mental health issues [21], noting that BPD was included in these studies. There is emerging evidence that telehealth can be utilised as an effective way to deliver DBT. One pilot study, conducted prior to COVID‐19, found participants reported the convenience of the online DBT group outweighed any negative effects [22]. There are a number of studies that have focused on clinician experiences of delivering DBT over telehealth (internationally and with a focus on Australia and New Zealand), with COVID‐19 as a catalyst. These papers include reflections on the challenges as well as guidance for how to provide quality care and overcome obstacles associated with telehealth [23]. Whilst some clinicians may worry about attendance, there is evidence to suggest no difference in rates of attendance when DBT was delivered via telehealth compared with face‐to‐face delivery [24]. Whilst this data is promising, to date there is very little published data regarding the treatment outcomes of DBT delivered over telehealth compared to face‐to‐face delivery. Research into DBT delivery also appears to have focused either on exclusively face‐to‐face treatment or telehealth treatment (in the wake of the COVID‐19 pandemic), but not an integrated, purposeful model of the two delivery formats. At the time of this paper, the authors are not aware of published evidence for collaborative models of DBT delivery where online and face‐to‐face components have been integrated to meet the needs of rural community health care.

## 5 A New Rural Workforce Model to Deliver DBT Treatment to People With BPD in Rural Communities

In addition to the use of telehealth, the second solution proposed in the Orange Declaration to address poor rural mental health outcomes is to develop new rural workforce models [15]. In this paper, we outline a potential new model of service delivery in rural settings that could involve collaborative delivery of DBT across rural mental health teams, rather than singular mental health teams delivering the whole program face to face. In our experience, DBT programs in singular rural mental health teams have had difficulty being sustained, due to DBT being resource intensive and rural mental health teams generally being small with multiple demands. This model would utilise telehealth and a new rural workforce model involving collaboration across rural mental health teams (and potentially metropolitan mental health teams) such that the skills group would be delivered virtually to consumers across the rural areas of a health service, with the individual therapy delivered face to face with local mental health clinicians. The DBT team would consist of therapists from across the health service who would meet virtually.

The new model of service delivery for DBT suggests providing a comprehensive 12‐month DBT program to consumers with BPD living in rural areas across a local health district (LHD) with some minor adaptations to standard DBT that has been evaluated in numerous research trials [11]. The differences in the new model of service delivery from Standard DBT are shown in Table 1. There are multiple benefits to the new suggested model. The clinicians and eligible consumers would be recruited from multiple rural sites within the Local Health District to work together. Recruiting across multiple sites also ensures that there would be enough clinicians to recruit between local sites to provide face‐to‐face care to their corresponding consumers. For example, a clinician in town A could provide face‐to‐face individual therapy to the consumer in town A, whereas a clinician in town B could provide therapy to the consumer in town B. This could be one way of trying to address chronic understaffing in rural settings by helping local rural sites to work together to create a DBT team across the Local Health District. This also ensures that local clinicians with shared rural experiences can provide good quality care to local consumers (e.g., instead of metropolitan clinicians providing exclusively telehealth services without a deep understanding of the experiences of living rurally). It is hoped that this may help reduce turnover of staff in rural areas by helping them to feel supported and that they are delivering effective care to this group of consumers. For the consumer, DBT commences with a pre‐treatment phase, which is provided by the individual therapist. There is no existing research to date regarding dropout when DBT treatment is delivered via telehealth; however, clinicians have expressed concern that it may lead to higher dropout [22]. As the pre‐treatment phase would hopefully be provided face to face (except for rural consumers where the travel is prohibitive), it allows the individual therapist to engage the consumer prior to beginning the group delivered via telehealth. By recruiting across multiple rural sites, there would be sufficient consumers to form a viable DBT group (DBT typically has a group of up to 8 consumers) and the consumers who join skills group would be granted greater anonymity in a rural setting.

**TABLE 1:** Differences in the new model of service delivery to provide DBT in rural community mental health settings compared with standard DBT.

Mode of therapy	Standard DBT (as per current published research)	New model of service delivery	Rationale for suggested change
Individual therapy	Delivered face to face	Delivered face to face by local rural clinicians (unless virtual is indicated)	Keeping to the model that has extensive research (i.e., face‐to‐face delivery) [11] whilst acknowledging some rural consumers may live hours away from their local rural community health team and require telehealth.
Skills training group	Face to face in one room	Delivered virtually	Allows participants to obtain benefit of group participation, which is often difficult/impossible in rural areas due to small numbers of clinicians and multiple demands on their time as well as distances for consumers.
Therapist consultation team	Consultation teams occur face to face with therapists who are co‐located in same physical space	Consultation teams occur virtually with therapists attending from their location across a health service	Running DBT programs in single rural teams is often prohibitive due to small teams and multiple demands. This model would allow therapists to come together from different rural teams and include experienced therapists from other locations, as required.
Phone consultation	Provided via phone calls or text	Provided via phone calls or text	This mode of the therapy has always been delivered in a virtual way and not face to face, and hence, no change.

Whilst there are some adaptations in the new suggested model to the standard comprehensive face‐to‐face DBT program, its design maintains as much fidelity to the original treatment model as possible. At this point in time, all of the published studies on DBT outcomes are based on face‐to‐face treatment delivery. Hence, we suggest maintaining the face‐to‐face components of treatment delivery where possible, but utilisation of a model that includes the use of telehealth is better than accepting no access to evidence‐based treatment at all in rural areas.

In this new model, clinicians would attend a weekly online DBT consultation team meeting (a form of group supervision) to provide support to each other and their consumers across the region. Clinicians often report lacking confidence working with this population and stress regarding managing suicide risk [8]. The weekly consultation meeting would help rural clinicians to develop their confidence and skills. Online group therapy could be delivered by local rural clinicians (if they had capacity within their caseload) or by metropolitan clinicians with expertise in DBT as the skills group is delivered via telehealth. These local clinicians or metropolitan health service clinicians would also join weekly consultation team meetings and be part of the rural DBT team and support network for the clinicians. If the group therapists are metropolitan clinicians with expertise, then this is another pathway to increasing confidence and skills of rural clinicians by partnering with experienced clinicians in metropolitan areas.

One challenge for any rural model of service delivery is the feasibility of rural clinicians providing regular psychotherapy amongst the multiple demands on clinicians in small teams. Services may consider developing new position descriptions and recruiting to specified DBT positions or adjusting caseloads for those clinicians who are involved in the DBT programs. We invite comment on the suggested model of service delivery put forward that is hoped to make DBT accessible in rural areas, including the degree to which this model is seen as acceptable, appropriate and feasible.

## 6 Conclusion

In summary, BPD is a serious mental health condition for which there are effective treatments. However, the common challenges faced by rural mental health care services coupled with the intensity and style of comprehensive DBT treatment have meant that delivering DBT in rural areas is not usually feasible. Telehealth and collaborative approaches to service delivery may offer a solution to this. If feasible, appropriate and acceptable, a model such as the one outlined in this paper would help reduce inequity of mental health care for people with BPD in rural areas and support the clinicians who treat them.

### Author Contributions

**Carla J Walton:** supervision, conceptualization, methodology, visualization, writing – review and editing. **Sharleen Gonzalez:** conceptualization, visualization, writing – original draft. **Anna Dunbar:** supervision, conceptualization, methodology, writing – review and editing. **Katie McGill:** conceptualization, methodology, supervision, visualization, writing – review and editing.

## Conflicts of Interest

C.J.W. is Service Director of Centre for Psychotherapy, a specialist service within Hunter New England Local Health District, based in Newcastle for the treatment of BPD. A.D. is the Deputy District Manager, Community Mental Health, Drug and Alcohol of Murrumbidgee Local Health District. Both hold responsibility for managing service quality and safety, within their respective services. K.M. has received Burdekin review funding. C.J.W. is a director and trainer for DBT Training Australia and provides training and consultation in DBT.

## Data Availability

The authors have nothing to report.
